# Effect of metformin and lifestyle intervention on adipokines and hormones in breast cancer survivors: a pooled analysis from two randomized controlled trials

**DOI:** 10.1007/s10549-023-07241-2

**Published:** 2024-01-26

**Authors:** Harriet Johansson, Federica Bellerba, Debora Macis, Bjørn-Erik Bertelsen, Aliana Guerrieri-Gonzaga, Valentina Aristarco, Kristin Viste, Gunnar Mellgren, Giulia Di Cola, Jemos Costantino, Augustin Scalbert, Dorothy D. Sears, Sara Gandini, Andrea DeCensi, Bernardo Bonanni

**Affiliations:** 1 IEO, European Institute of Oncology IRCCS, Milan, Italy; 2 Hormone Laboratory, Department of Medical Biochemistry and Pharmacology, Haukeland University Hospital, Bergen, Norway; 3 Department of Clinical Science, University of Bergen, Bergen, Norway; 4 International Agency for Research on Cancer, Nutrition and Metabolism Branch, Lyon, France; 5 College of Health Solutions, Arizona State University, Phoenix, AZ USA; 6 Moores Cancer Center, UC San Diego, La Jolla, CA USA; 7 Department of Medicine, UC San Diego, La Jolla, CA USA; 8 Department of Medicine and Medical Oncology, E.O. Ospedali Galliera, Genoa, Italy; 9 Wolfson Institute of Population Health, Queen Mary University of London, London, UK

**Keywords:** Weight loss, Recurrence biomarkers, Sex hormones, Inflammation, Cytokines

## Abstract

**Purpose:**

We investigated the effect of metformin and lifestyle intervention on metabolic, inflammatory, and steroid biomarkers of breast cancer (BC) recurrence risk in two intervention trials among BC survivors with overweight or obesity.

**Methods:**

Baseline and follow-up serum samples collected during the two trials were analyzed and data pooled. The USA trial (Reach for Health) included postmenopausal BC survivors (n = 333) randomly assigned to 6-month metformin vs placebo and lifestyle intervention (LSI) vs control (2 × 2 factorial design). The Italian trial (MetBreCS) included BC survivors (n = 40) randomized to 12-month metformin vs placebo. Insulin resistance (HOMA-IR), adipokines, cytokines, and steroids were measured.

**Results:**

Metformin compared to placebo showed a favorable decrease in leptin (− 8.8 vs − 3.5 ng/mL; p < 0.01) and HOMA-IR (− 0.48 vs − 0.25; p = 0.03), and an increase in SHBG (2.80 vs 1.45 nmol/L; p < 0.01). Excluding women taking aromatase inhibitors, metformin (n = 84) compared to placebo (n = 99) decreased estradiol (− 4 vs 0 pmol/L; p < 0.01), estrone (− 8 vs 2 pmol/L; p < 0.01) and testosterone (− 0.1 vs 0 nmol/L-; p = 0.02). LSI favorably affected adiponectin (0.45 vs − 0.06 ug/mL; p < 0.01), leptin (− 10.5 vs − 4.4 ng/mL; p < 0.01), HOMA-IR (− 0.6 vs 0.2; p = 0.03), and SHBG (2.7 vs 1.1 nMol/L; p = 0.04) compared to controls. The strongest impact was observed combining metformin with LSI on adipokines, CRP, SHBG, and estrogens.

**Conclusions:**

Supportive healthy lifestyle programs combined with metformin to achieve maximal risk reduction among BC cancer survivors are recommended, especially for those with obesity in menopause.

**Supplementary Information:**

The online version contains supplementary material available at 10.1007/s10549-023-07241-2.

## Introduction

In Western countries, over-nutrition has become a vast challenge to deal with. Insulin resistance and compensatory hyperinsulinemia occur as a consequence of nutrient overload and associated adipocyte hypertrophy and adipose tissue inflammation. Obesity and insulin resistance are important modifiable risk factors associated with breast cancer incidence, recurrence, and worse overall survival, despite the administration of appropriate local and systemic therapies [1–3].

Elevated breast cancer risk associated with increasing BMI among postmenopausal women is related to an increase in estrogens, which are generated by increased aromatase in adipose tissue [4]. After menopause, when the ovary activity slows down, estrogens are mainly generated by peripheral aromatization of androgens originating from the adrenals or ovaries [5]. Estrogens might affect breast carcinogenesis through their oxidative metabolites and by affecting cell proliferation through their interaction with the estrogen receptors (ER) in breast tissue [6].

Obesity also enhances cancer risk via systemic metabolic effects such as insulin resistance which is associated with tumor-promoting hyperinsulinemia and hyperglycemia [7]. Adipokine secretion, hyperinsulinemia, estrogen signaling, and inflammation play important roles in promoting breast cancer progression, activating the PI3K/AKT/mTOR pathway [8]. Metformin is a biguanide used as a first-line treatment for type 2 diabetes that inhibits hepatic gluconeogenesis and sensitizes insulin action in the peripheral tissues. Metformin reduces diabetes incidence in obese women with glucose intolerance with a good tolerability profile [9]. Metformin has been shown to improve insulin sensitivity and reduce circulating insulin in nondiabetic breast cancer subjects [10]. The drug has been proposed to have anti-cancer activities by acting directly on tumor cell metabolism, in particular through the inhibition of oxidative phosphorylation of tumor cell mitochondria, and by acting as a PI3K-Akt-mTOR pathway inhibitor. Metformin has been associated with better survival in patients with breast cancer treated for diabetes, compared to other diabetic drugs [11, 12] although a phase III trial showed a lack of efficacy of metformin in preventing breast cancer recurrence in non-diabetic women except for the subgroup of HER-2 positive disease [13].

Over two-thirds of breast cancer survivors are overweight or obese and do not meet physical activity guidelines. Changes in systemic hormonal, cytokine, adipokine, and insulin pathway biomarkers influenced by weight loss and physical activity may help define both the necessary weight loss and the influence of regain on the likelihood of cancer recurrence. This trend can be slowed down by dietary approaches, increasing physical activity as well as adopting drug strategies to prevent or reduce hyperinsulinemia early, before obesity advances.

The purpose of this combined analysis was to examine the impact of metformin and lifestyle intervention on biomarkers implicated in recurrence in breast cancer survivors at high risk for recurrence because of unhealthy lifestyle, adiposity, or breast cancer subtype. Biomarkers related to insulin resistance and inflammatory pathways as well as a panel of eight steroid hormones were measured.

## Methods

### Study design

Within a tertiary prevention trial, we investigated the mechanisms underlying the activity of metformin on metabolic impairment with a focus on estrogen receptor-negative disease. Because the trial did not reach the desired sample size, we combined data with the Reach for Health trial (RFH) trial [14].

### Brief description of the two trials

The RFH was approved by the Human Research Protections Program at UC San Diego (ClinicalTrials.gov identifier: NCT01302379), and participants signed informed consent forms [14]. Briefly, postmenopausal breast cancer survivors with overweight or obesity (n = 333; BMI ≥ 25.0 kg/m^2^) were randomly assigned to a 6-month treatment with metformin versus placebo and lifestyle intervention vs control in a 2 × 2 factorial fashion. Metformin vs Placebo arms included participants randomly assigned to receive metformin or placebo pills. Participants were further randomly assigned to a telephone-based weight loss intervention or control.

The Italian MetBreCS trial (EudraCT Protocol #: 2015-001001-14), a mono-institutional, randomized placebo-controlled phase II study for breast cancer survivors with BMI ≥ 25.0 kg/m^2^ (n = 40) at higher risk for recurrence (TNBC, non-luminal HER2+, and Luminal B HER2+) was approved by the local IRB at IEO, Milan, Italy, and participants signed informed consent.

### Serum biomarkers of insulin resistance

Serum adiponectin, leptin, resistin, complement factor D, monocyte chemoattractant protein 1 (CCL2), Serpin (PAI-1), IL-6, IL-10, TNF-alpha (TNF-a) were measured using an automated immunoassay platform called ELLA (ProteinSimple, Bio-techne, Minneapolis, MN, USA) [15].

Serum concentrations of IGF-I, IGFBP-3, and SHBG were determined by a chemiluminescent immunoassay designed for the IDS-iSYS Multi-Discipline Automated System (Immunodiagnostic Systems Limited, United Kingdom).

Serum concentrations of insulin were determined by a chemiluminescent microparticle immunoassay, performed on the automated instrument ARCHITECT i System (Abbott Laboratories, Wiesbaden, Germany). Serum concentrations of C-reactive protein were determined by an immunoturbidimetric assay using the automated instrument ALINITY c analyzer (Abbott Laboratories, Wiesbaden, Germany). HOMA-IR [fasting insulinemia (mU/L) x glycemia (mmol/L)]/22.5 was applied as a surrogate index of insulin resistance.

### Steroid biomarkers

Steroid hormones were measured in 709 patient samples (Hormone Laboratory, Haukeland University Hospital, Bergen Norway). Serum samples were analyzed for estradiol, estrone, cortisol, testosterone, androstenedione, progesterone, 11-deoxycortisol, and 17-hydroxyprogesterone, mapping large parts of the steroid hormone synthesis pathway.

Estradiol and estrone were analyzed using an ultrasensitive and thoroughly validated LC–MS/MS method [16]. All steps were fully automated. Because of the low sample volume, most samples had to be diluted (1 part of patient serum, 3 parts steroid-depleted human serum), thus increasing the functional LLOQ to 1.7 pmol/L and 0.9 pmol/L for estradiol and estrone, respectively. Results below LLOQ were assigned an arbitrary value of LLOQ/2, *i.e.* estradiol 0.85 pmol/L and estrone 0.45 pmol/L [17]. LLOQs were still well below the reference range for estradiol and estrone in postmenopausal women [16].

Testosterone, progesterone, cortisol, androstenedione, 17-hydroxyprogesterone, and 11-deoxycortisol were measured by a previously described multi-steroid LC–MS/MS assay [18]. The method is included in the NEQAS program and all measurement ranges cover the expected levels for postmenopausal women.

### Statistical methods

Descriptive statistics are presented by trial arm including tumor characteristics of the participants and baseline median values and interquartile ranges of serum biomarkers, applying a 2-sided P-value to evaluate differences among arms (Kruskal–Wallis test for continuous variables and Chi-square test for categorical variables). We also presented median values and interquartile ranges of baseline and absolute changes of serum biomarkers by treatment (metformin vs placebo) and lifestyle intervention (LSI) groups (yes vs no), collapsing the original trial arms. The effects of metformin and LSI on the serum biomarkers changes were evaluated through ANCOVA models adjusted for baseline values, study center, age, baseline BMI and aromatase inhibitor therapy (AI). Least square means from models including the four types of intervention (placebo, metformin, LSI, LSI + metformin) as a covariate are also presented to investigate a potential additive effect of LSI on that of metformin only (LSI + metformin). We carried out the same analyses for the subgroups of women not taking AIs and for postmenopausal women not taking AIs in regard to steroids (namely estrone, estradiol, and testosterone), and according to estrogen receptor status in regard to absolute changes of serum biomarkers (adiponectin, leptin, and SHBG). The normal distribution of residuals from full models was graphically checked. When the normality assumption was not met, extreme outliers were excluded from the models. All analyses were carried out using R statistical software, version 4.1.2. Spearman rank’s correlation coefficient between baseline inflammatory and metabolic biomarkers and BMI was performed.

## Results

**Fig. 1:**
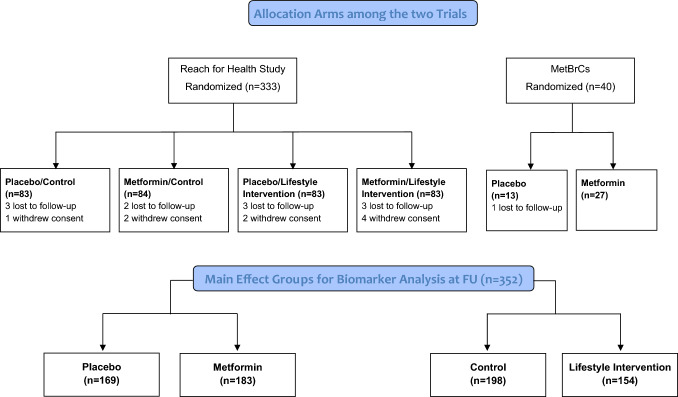
Flow diagram showing study design of the two trials and main effect comparisons for biomarker pooled analysis

A flow diagram showing the treatment allocation of the two trials and main effect comparisons are shown in Fig. 1. Participants taking metformin were pooled together against those taking placebo pills, irrespective of lifestyle intervention. Participants randomized to lifestyle intervention were pooled together against participants not enrolled in the lifestyle intervention. A blood draw was performed at baseline and at treatment termination, namely 6 months in the RFH trial and 12 months in the MetBreCS trial. A total of 373 women were randomized, and 352 participants had follow-up serum samples available.

### Participant characteristics

Baseline host and tumor characteristics of participants according to the allocation arm have been published [19]. Several breast cancer characteristics at diagnosis that are associated with increased risk of recurrence were more frequent in the Italian MetBreCS trial, such as grading, hormone receptor, and HER2 status were more common in the MetBreCS trial as this trial was designed for breast cancer survivors at high risk of recurrence. Mean BMI was greater in the RFH study population and a great proportion of US study women (57%) were taking aromatase inhibitors (Supplementary Table S1).

Baseline median and interquartile ranges of circulating biomarkers of insulin resistance by allocation arm are shown in Supplementary Table S2. We observed some differences in baseline biomarkers between the two trials, which may at least in part be attributable to the statistically significant differences in BMI between the two cohorts [19]. Overall, 54% of women in RFH were obese versus 35% of the MetBreCS cohort Insulin, HOMA index, complement factor D, and IL-10 were statistically significantly higher in the RFH trial. At variance with the RFH, the MetBreCS women were younger and had higher levels of PAI-I, plausibly attributable to the presence of premenopausal women (41%), known to have higher concentrations of PAI-I than postmenopausal women [20].

Baseline median and interquartile ranges of circulating steroids by allocation arm are shown in Supplementary Table S3. The RFH trial included women still taking endocrine adjuvant treatment, while the MetBreCS trial only included women who had completed their adjuvant treatment. In the table, we report the frequency of women taking aromatase inhibitor therapy as this drastically affects estrogen levels. Women were asked to maintain the same adjuvant treatment during the whole study duration. In postmenopausal women treated with aromatase inhibitors, very low to undetectable levels of estrogens are expected [16]. This explains the statistically significant difference in baseline estradiol and estrone levels between the two trials. Results below the lower limit of quantification (LOQ) were assigned an arbitrary value of LOQ/2 [17] *i.e.,* estradiol 0.85 pmol/L and estrone 0.45 pmol/L. Thus, regarding these two steroids, we describe both overall results and results from the subgroup of women not taking aromatase inhibitors.

### Biomarker changes upon metformin and lifestyle intervention

Changes in circulating biomarkers of insulin resistance and steroids not affected by aromatase inhibitors are presented in Table 1 as main effect groups. After adjustments for confounders, metformin treatment compared to placebo was significantly associated with a favorable decrease in circulating leptin (− 8.8 *vs* − 3.5 ng/mL; p < 0.01), insulin, and HOMA-index (− 0.48 *vs* − 0.25; p = 0.03), and an increase in adiponectin/leptin ratio and SHBG (2.80 vs 1.45 nmol/L; p < 0.01) levels. Lifestyle intervention (Table 2) showed a strongly favorable effect on both adipokines, as evidenced by a marked increase in adiponectin (0.45 vs − 0.05 ug/mL; p < 0.01), as well as a steep decrease in leptin levels (− 10.5 vs − 4.4 ng/mL; < 0.01). Insulin levels and HOMA-index (− 0.6 vs 0.2; p = 0.03) also decreased, and SHBG levels increased (2.7 vs 1.1 nmol/L; p = 0.04). No significant modulation in other markers of adiposity and inflammatory markers was observed, neither with metformin nor by LSI.

**Table 1:** Baseline median and interquartile ranges of serum biomarkers and absolute change at follow-up by metformin

	Placebo		Metformin		p value of metformin
	Baseline	Absolute change	Baseline	Absolute change	
Adiponectin (ug/mL)	10.3 [7.54, 13.3]	0.080 [− 0.82, 0.90]	10.3 [6.96, 14.0]	0.130 [− 0.975, 1.16]	0.68
Leptin (ng/mL)	44.3 [31.2, 66.1]	− 3.48 [− 14.9, 6.35]	45.2 [31.0, 67.7]	− 8.81 [− 20.9, 0.135]	** < 0.01**
Adiponectin/leptin	0.23 [0.145, 0.37]	0.010 [− 0.03, 0.13]	0.225 [0.13, 0.35]	0.06 [− 0.01, 0.22]	** < 0.01**
Insulin (uU/mL)	11.4 [8.41, 15.6]	− 1.04 [− 3.69, 1.68]	10.9 [7.97, 16.9]	− 1.64 [− 4.38, 0.600]	**0.02**
HOMA-IR	2.88 [2.03, 4.03]	− 0.250 [− 0.970, 0.520]	2.67 [1.97, 4.00]	− 0.480 [− 1.14, 0.180]	**0.03**
IGF-I (ng/mL)	121 [97.1, 148]	− 3.63 [− 14.1, 10.4]	121 [98.3, 156]	− 3.39 [− 18.4, 9.39]	0.34
IGFBP-3 (ug/mL)	3.82 [3.29, 4.34]	− 0.0200 [− 0.310, 0.200]	3.94 [3.35, 4.37]	− 0.140 [− 0.320, 0.165]	0.21
SHBG (nmol/L)	43.9 [33.5, 60.8]	1.45 [− 3.50, 6.15]	44.4 [31.4, 63.4]	2.80 [− 1.85, 10.1]	** < 0.01**
CRP (mg/dL)	0.300 [0.150, 0.630]	0.0100 [− 0.100, 0.130]	0.300 [0.143, 0.635]	− 0.0400 [− 0.150, 0.0350]	0.27
IL-6 (pg/mL)	2.47 [1.86, 3.30]	0.120 [− 0.540, 0.610]	2.42 [1.78, 3.57]	− 0.120 [− 0.755, 0.530]	0.23
IL-10 (pg/mL)	1.71 [1.47, 2.21]	0.0400 [− 0.190, 0.200]	1.84 [1.53, 2.32]	0.0600 [− 0.240, 0.335]	0.19
TNFa (pg/mL)	8.58 [7.56, 9.76]	0 [− 0.680, 0.700]	8.51 [7.37, 9.86]	0.12 [− 0.62, 0.845]	0.80
CCL2 (pg/mL)	378 [295, 475]	− 12.0 [− 56.0, 40.0]	359 [276, 443]	6.00 [− 48.8, 47.8]	0.95
Complement factor D (ng/ml)	3460 [2970, 4070]	− 18.9 [− 208, 201]	3410 [2890, 3860]	17.2 [− 242, 209]	0.85
Resistin (ng/mL)	8.69 [7.01, 11.6]	0.130 [− 0.850, 0.930]	8.50 [6.91, 10.7]	0.270 [− 0.750, 1.38]	0.06
Serpin or PAI-1 (ng/mL)	114 [91.9, 143]	2.69 [− 14.6, 17.4]	106 [86.2, 131]	5.42 [− 9.80, 17.1]	0.27
11-deoxycortisol (nmol/L)	0.600 [0.400, 0.800]	0 [− 0.200, 0.200]	0.500 [0.300, 0.800]	0 [− 0.200, 0.200]	0.08
17-hydroxyprogesterone (nmol/L)	0.400 [0.300, 0.600]	0 [− 0.100, 0.200]	0.400 [0.300, 0.600]	0 [− 0.100, 0.175]	0.32
Androstendione (nmol/L)	1.40 [1.10, 2.00]	0 [− 0.300, 0.300]	1.50 [1.00, 1.90]	0 [− 0.275, 0.200]	0.41
Cortisol (nmol/L)	244 [200, 303]	5.00 [− 42.0, 65.0]	238 [186, 313]	34.0 [− 24.0, 82.0]	0.35

p value of the treatment covariate (Metformin vs Placebo) derived from a multivariate linear model fit on biomarker changes (Follow-up visit—Baseline), adjusted for the baseline value of the biomarker, study center, lifestyle intervention, age, baseline BMI and aromatase inhibitor therapy

Bold characters in table was adopted for p-values below 0.05

**Table 2:** Baseline median and interquartile ranges of serum biomarkers and absolute change at follow-up by lifestyle intervention (LSI)

	LSI no		LSI yes		p value of LSI
	Baseline	Absolute change	Baseline	Absolute change	
Adiponectin (ug/mL)	10.2 [7.27, 13.6]	− 0.0550 [− 1.05, 0.680]	10.4 [7.16, 13.9]	0.450 [− 0.605, 1.52]	** < 0.01**
Leptin (ng/mL)	42.2 [30.0, 66.1]	− 4.39 [− 15.3, 7.05]	46.6 [32.0, 67.8]	− 10.5 [− 22.9, − 1.89]	** < 0.01**
Adiponectin/leptin	0.24 [0.145, 0.37]	0 [− 0.030, 0.118]	0.22 [0.13, 0.338]	0.075 [0.01, 0.295]	** < 0.01**
Insulin (uU/mL)	9.83 [7.89, 14.9]	− 0.945 [− 3.04, 1.68]	12.2 [8.52, 17.3]	− 2.45 [− 5.05, 0.460]	** < 0.01**
HOMA-IR	2.51 [1.93, 3.85]	0.235 [− 0.878, 0.495]	2.97 [2.23, 4.60]	− 0.605 [− 1.48, 0.168]	**0.03**
IGF-I (ng/mL)	124 [99.9, 156]	− 4.44 [− 16.4, 10.4]	118 [95.3, 146]	− 2.19 [− 13.8, 8.88]	0.78
IGFBP-3 (ug/mL)	3.97 [3.36, 4.39]	− 0.0550 [− 0.320, 0.200]	3.78 [3.30, 4.25]	− 0.110 [− 0.300, 0.138]	0.94
SHBG (nmol/L)	43.9 [32.6, 60.9]	1.10 [− 3.08, 6.44]	44.0 [33.3, 63.0]	2.70 [− 2.33, 10.7]	**0.04**
CRP (mg/dL)	0.295 [0.150, 0.608]	0.0200 [− 0.140, 0.0900]	0.320 [0.150, 0.650]	− 0.0100 [− 0.140, 0.0600]	0.07
IL-6 (pg/mL)	2.39 [1.80, 3.40]	− 0.0450 [− 0.758, 0.610]	2.54 [1.78, 3.42]	0.0850 [− 0.525, 0.513]	0.43
IL-10 (pg/mL)	1.76 [1.47, 2.25]	0.0650 [− 0.218, 0.310]	1.78 [1.49, 2.31]	0.0250 [− 0.240, 0.248]	0.45
TNFa (pg/mL)	8.55 [7.39, 9.71]	0.07 [− 0.633, 0.818]	8.48 [7.44, 10.0]	0.09 [− 0.645, 0.713]	0.84
CCL2 (pg/mL)	378 [287, 459]	− 4.00 [− 56.0, 38.0]	361 [283, 457]	3.50 [− 49.8, 67.8]	0.10
Complement factor D (ng/mL)	3290 [2890, 3860]	33.9 [− 198, 234]	3610 [3080, 4140]	− 53.2 [− 278, 167]	0.46
Resistin (ng/mL)	8.46 [7.03, 10.5]	0.285 [− 0.728, 1.33]	8.66 [6.70, 11.5]	0.120 [− 0.833, 0.978]	0.66
Serpin or PAI-1 (ng/mL)	118 [96.5, 146]	5.98 [− 11.6, 16.3]	99.5 [80.7, 124]	3.07 [− 13.2, 17.7]	0.96
11-deoxycortisol (nmol/L)	0.500 [0.300, 0.800]	0 [− 0.200, 0.200]	0.500 [0.400, 0.800]	0 [− 0.200, 0.200]	0.98
17-hydroxyprogesterone (nmo1/14	0.400 [0.300, 0.600]	0 [− 0.100, 0.200]	0.400 [0.300, 0.600]	0 [− 0.100, 0.100]	0.30
Androstendione (nmol/L)	1.50 [1.00, 1.90]	0 [− 0.300, 0.300]	1.40 [1.10, 1.90]	0 [− 0.200, 0.275]	0.34
Cortisol (nmol/L)	244 [188, 314]	14.0 [− 39.0, 85.0]	237 [195, 300]	18.0 [− 34.0, 66.0]	0.77

p value of the lifestyle intervention (LSI) covariate (Yes vs No) derived from a multivariate linear model fit on biomarker changes (Follow-up visit—Baseline), adjusted for the baseline value of the biomarker, treatment, study center, age, baseline BMI and aromatase inhibitor therapy

Bold characters in table was adopted for p-values below 0.05

The changes in estrogen and testosterone are presented in Table 3. We decided to investigate the effect of metformin and lifestyle intervention only in the subgroup of women not taking aromatase inhibitors, due to the strong suppression observed at baseline. Metformin (n = 84) compared to placebo (n = 99) was associated with a decrease in estradiol (-4 *vs* 0 pmol/L; p < 0.01), estrone (− 8 *vs* 2 pmol/L; p < 0.01) and testosterone (− 0.1 *vs* 0 nmol/L-; p = 0.02), while no significant effects by lifestyle intervention were observed. The same results were confirmed after excluding the premenopausal women (Supplementary Table S3).

**Table 3:** Baseline median and interquartile ranges of serum steroids in women not taking AIs and absolute change at follow-up by main effect groups

	Placebo (n = 84)		Metforrnin (n = 99)		p metformin*	LSI no (n = 107)		LSI yes (n = 76)		p lifestyle**
	Baseline	Absolute change	Baseline	Absolute change		Baseline	Absolute change	Baseline	Absolute change	
Estradiol (pmol/L)	19.0 [13.5, 29.5]	0 [− 2.50, 3.00]	20.0 [13.0, 30.0]	− 4.00 [− 8.50, 0]	** < 0.01**	20.0 [14.0, 28.8]	− 1.00 [− 5.80, 1.83]	20.0 [13.0, 30.8]	− 0.800 [− 7.03, 1.00]	0.84
Plasma sample missing	9 (10.7%)	9 (10.7%)	14 (14.1%)	16 (16.2%)		13 (12.1%)	13 (12.1%)	10 (13.2%)	12 (15.8%)	
Estrone (pmol/L)	97.0 [71.0, 140]	2.00 [− 6.50, 13.0]	98.0 [62.0, 130]	− 8.00 [− 29.0, 2.00]	** < 0.01**	96.5 [64.0, 126]	− 1.50 [− 18.8, 9.50]	101 [66.0, 140]	− 4.00 [− 24.8, 5.25]	0.26
Plasma sample missing	9 (10.7%)	9 (10.7%)	14 (14.1%)	16 (16.2%)		13 (12.1%)	13 (12.1%)	10 (13.2%)	12 (15.8%)	
Testosterone (nmol/L)	0.600 [0.500, 0.800]	0 [− 0.100, 0.100]	0.600 [0.400, 0.800]	− 0.100 [− 0.100, 0]	**0.02**	0.600 [0.400, 0.700]	0 [− 0.100, 0.100]	0.600 [0.400, 0.800]	− 0.100 [− 0.100, 0]	0.52
Plasma sample missing	11 (13.1%)	12 (14.3%)	14 (14.1%)	15 (15.2%)		13 (12.1%)	13 (12.1%)	12 (15.8%)	14 (18.4%)	

*p value of the treatment covariate (Metformin vs Placebo) derived from a multivariate linear model fit on biomarker changes (Follow-up visit—Baseline), adjusted for the baseline value of the biomarker, study center, lifestyle intervention (LSI), age and baseline BMI

**p value of the LSI covariate (Yes vs No) derived from a multivariate linear model fit on biomarker changes (Follow-up visit—Baseline), adjusted for the baseline value of the biomarker, treatment, study center, age and baseline BMI

Bold characters in table was adopted for p-values below 0.05

**Fig. 2:**
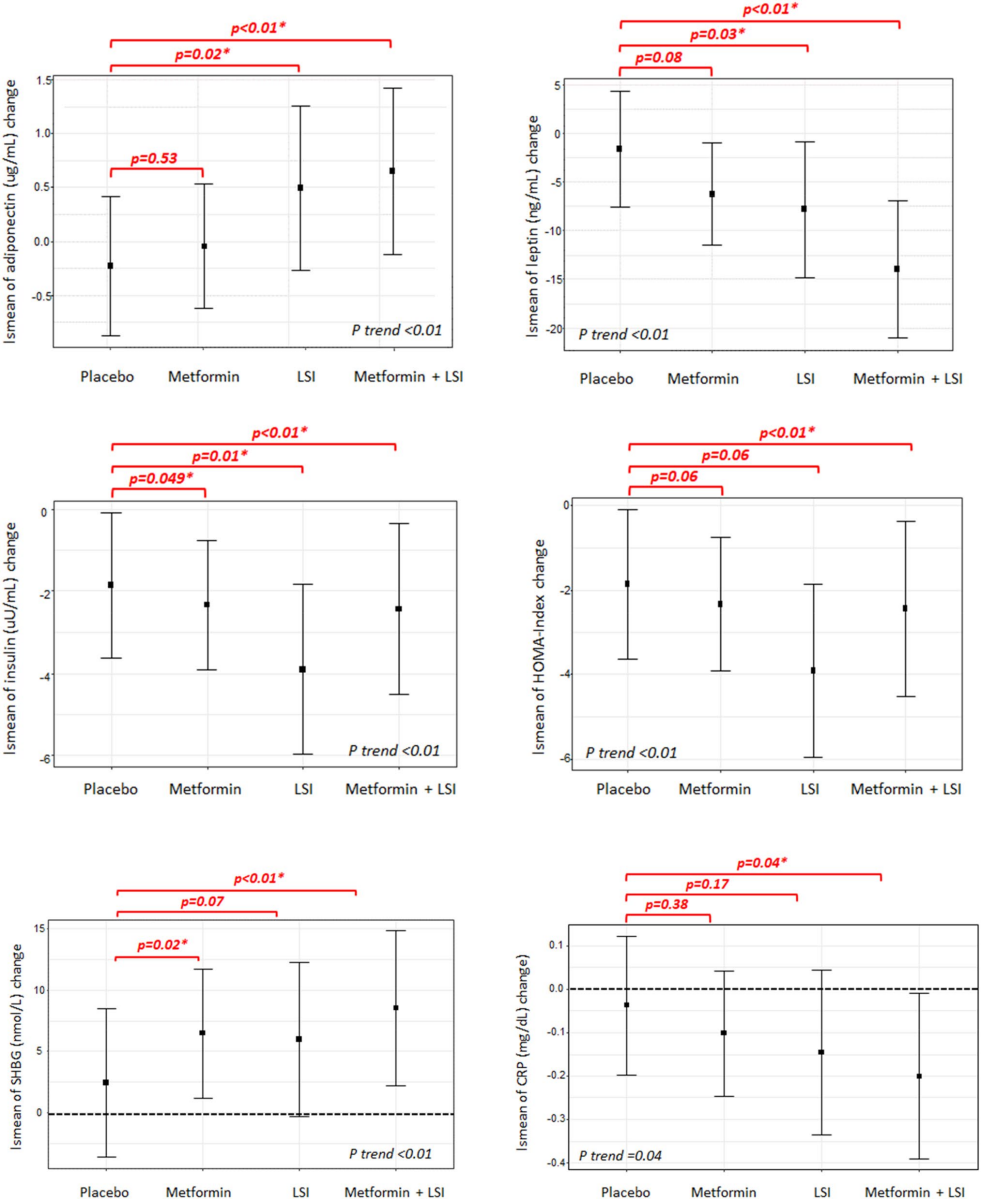
Exploratory analysis of an additive effect of metformin with lifestyle intervention by least square mean changes through the four main effect groups. Least-square means derived from multivariable linear regression models fit on biomarker changes (Time 2—time 1), adjusted for the baseline value of the biomarker, study center, age, aromatase inhibitor therapy and baseline BMI. The treatment effect on biomarker changes was assessed by including a 4-level categorical variable as a covariate, with each level corresponding to one of the four types of intervention planned in the two cohorts (placebo, metformin, LSI, metformin + LSI). The p values referring to the comparison of each intervention level versus placebo are indicated as p, which was taken as reference. P trend is the p value referring to the trend effect of the treatment, assuming the following intervention intensity scale: placebo, metformin, LSI, metformin + LSI

**Fig. 3:**
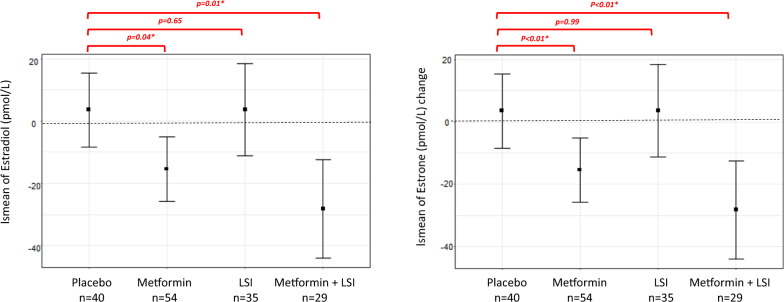
Exploratory analysis of an additive effect of metformin with lifestyle intervention by least square mean changes through the four main effect groups. Least-square means derived from multivariable linear regression models fit on biomarker changes (Time 2—time 1), adjusted for the baseline value of the biomarker, study center, age, aromatase inhibitor therapy and baseline BMI. The treatment effect on biomarker changes was assessed by including a 4-level categorical variable as a covariate, with each level corresponding to one of the four types of intervention planned in the two cohorts (placebo, metformin, LSI, metformin + LSI). The p values referring to the comparison of each intervention level versus placebo are indicated as p, which was taken as reference. P trend is the p value referring to the trend effect of the treatment, assuming the following intervention intensity scale: placebo, metformin, LSI, metformin + LSI

We explored the existence of an improved effect of metformin in combination with LSI, through the four main effect groups (Figs. 2, 3). The combination of metformin with lifestyle intervention lead to the strongest favorable impact on adipokines, SHBG, and CRP, maintaining the same consistent trend of all these biomarkers (Fig. 2). Metformin treatment was associated with favorable, significantly reduced levels of estradiol and estrone, compared to the placebo/control (Fig. 3). On average, this effect was enhanced in the combination intervention; LSI alone showed no effect. The analyses were adjusted for the baseline value of the biomarker, study center, age, and baseline BMI.

We also investigated any interaction of ER status at diagnosis (Supplementary Table S4) with biomarker changes. We found similar favorable metabolic effects of metformin and lifestyle intervention regardless of ER status at diagnosis.

In Supplementary Fig. 1 we describe Spearman correlations between adipokines, inflammatory cytokines, and BMI at baseline. Strong direct correlations between leptin and BMI (Spearman rank’s correlation coefficient: 0.638, p < 0.0001), and between CRP and IL-6 (0.58; p < 0.0001) were observed. Indirect correlations of the HOMA index with adiponectin/leptin ratio (− 0.54; p < 0.0001), as well as with SHBG (− 0.59; p < 0.0001), were observed. Moderate correlations between BMI and several inflammatory cytokines: CRP (0.40, p < 0.0001), IL-6 (0.384, p < 0.0001), and TNF-alpha (0.189, p < 0.0002).

## Discussion

Our findings in overweight to obese breast cancer survivors provide evidence of a favorable effect of metformin and lifestyle intervention on biomarkers involved in the breast carcinogenesis pathways. Apart from the known decrease in insulin levels and insulin resistance, one of the most marked changes was observed for leptin, which decreased with both metformin or lifestyle intervention, with a further improved effect in the combined treatment arm. Goodwin and colleagues [21] reported similar relative changes with metformin compared to placebo in their MA.32 trial, despite lower baseline leptin levels, in line with the lower median BMI in their trial. The WISER Survivor trial in overweight/obese breast cancer survivors observed significant decrements in leptin in the weight loss arms [22]. Adiponectin favorably increased only in the lifestyle intervention group. Leptin is an adipocyte-derived hormone strongly correlated with total subcutaneous body fat [23] that exerts powerful effects both centrally and peripherally [24]. In the brain, leptin inhibits food intake, promotes energy expenditure, and regulates autonomic nerve control, thus playing a key role in body weight regulation [25]. The hepatic leptin receptor was identified as a target gene being upregulated by metformin [26], which may enhance leptin sensitivity in the liver. Leptin also plays an important role in proinflammatory immune responses [27, 28].

During the development of obesity, adipose tissue macrophage infiltration increases [29]. The macrophages and adipocytes are the major TNFα and IL-6 sources in individuals with obesity. Together these cells are also involved in a feedback loop that perpetuates macrophage recruitment and production of proinflammatory cytokines [25, 30]. Conversely, metformin treatment decreased oxidative stress and mitochondrial dysfunction in adipose cells from abdominal subcutaneous fat obtained from healthy older women (> 60 years) [30]. Metformin has been described to improve chronic inflammation through its metabolic effects [21] but also by a direct anti-inflammatory impact locally [31]. The CRP decrease by metformin in our pooled analysis was not significant unless combined with LSI. Several studies indicate that metformin may influence IL-6 levels and ameliorate the state of chronic inflammation [32], but we, like others [33] were unable to confirm such an effect in our pooled analysis.

Overall, the impact of metformin was greater when combined with lifestyle intervention, as evidenced by a stronger change in adipokines, SHBG, and CRP serum levels in the combination arm. Moreover, these participants lost statistically more weight than those in the placebo group [14]. We acknowledge that the sample size estimates were based on main effects comparisons of metformin versus placebo and weight loss versus control. Thus, the sensitivity analysis exploring an improved effect of metformin in combination with lifestyle intervention is only suggestive and not conclusive. Indeed, these biomarkers are all strictly correlated with fat mass, insulin resistance, metabolic syndrome, pro-inflammatory state, and increased breast cancer risk [2–5, 7, 8, 34, 35]. Notably, neither metformin nor lifestyle intervention was able to reduce fasting plasma glucose levels in the RFH trial [14]. A large phase III adjuvant trial [13] concluded that metformin did not improve invasive disease-free survival.

Stronger metabolic changes might be required to obtain satisfactory long-term risk reductions. The strategy of combining metformin use with intermittent fasting/aerobic exercise or with other target drugs involved in the downstream pathway cascade toward tumorigenesis appears promising. Prolonged nightly fasting intervals of at least 13 h point to a reduction in breast cancer recurrence [36]. The combination of metformin with intermittent fasting, impaired tumor growth only when administered during fasting-induced hypoglycemia in a mice model [37]. A presurgical window of opportunity trial evaluating metformin and intermittent fasting on breast cancer growth is ongoing (ClinicalTrials.gov Identifier: NCT05023967).

The adipose tissue also regulates the production of sex hormones [38] which are considered to mediate the association of adiposity with breast cancer risk by expressing aromatase enzymes, and by increasing the bioavailability of free estradiol and testosterone, through hyperinsulinemia, elevated IGF-1 bioavailability, and decreased hepatic secretion of SHBG. In postmenopausal women, the rate of transformation of androgens to estrogens is higher amongst obese women [4, 38] and aromatase expression in the breast tissue is directly correlated with BMI and white adipose tissue inflammation [39]. An important difference between the cohorts was the use of adjuvant hormone therapy. Contrary to the RFH, where 57% of participants were taking aromatase inhibitors, in the MetBreCS trial women had concluded any adjuvant endocrine therapy before entering the trial. Notwithstanding, most women taking aromatase inhibitors had serum estrogen concentrations below the LLOQ. Thus, the metformin effects on estradiol, estrone, and testosterone were restricted to women not taking aromatase inhibitors (n = 183). Metformin significantly reduced all three sex steroids, showing a favorable effect on these breast cancer risk biomarkers [40, 41]. Similar effects of metformin were previously reported [42]. We recently observed in our metabolomics study of these samples, that metformin seems to increase the activity of the enzyme CYP1A2 [19]. This enzyme catabolizes estradiol and women with genomic variants of the CYP1A2 gene with less enzymatic activity may be at increased risk [43].

Another piece of evidence from our pooled analysis was the result of similar favorable metabolic effects of metformin and lifestyle intervention, regardless of ER status at diagnosis. A recent pooled analysis confirms the associations between modifiable lifestyle factors and 10-year all-cause mortality, without any strong evidence of associations by ER status or intrinsic-like subtype [44].

In summary, our pooled analyses show that metformin and lifestyle intervention advantageously affected adipokines, insulin resistance, inflammation, and sex steroid bioavailability, with the strongest impact in the combination arm. These findings hold the potential for a reduction in BC recurrence with a supportive healthy lifestyle alongside chemoprevention to achieve maximal risk reduction, especially for obese women after menopause.

### Supplementary Information

Below is the link to the electronic supplementary material.

Supplementary file1 (DOCX 62 KB)

Supplementary file2 (DOCX 350 KB)

## Data Availability

The data underlying this article may be shared upon reasonable request to the PI of the MetBrCs study (Dr Bernardo Bonanni), following approval by the Data and Safety Monitoring Board at IEO, Milan.
