# Supplementary material for: Effect of metformin and lifestyle intervention on adipokines and hormones in breast cancer survivors: a pooled analysis from two randomized controlled trials
Source: Breast Cancer Res Treat. 2024 Jan 26;205(1):49–59. doi: 10.1007/s10549-023-07241-2 (PMC11063007; doi:10.1007/s10549-023-07241-2)
Supplement: Supplementary file 1 — Supplementary file1 (DOCX 62 KB) [file 10549_2023_7241_MOESM1_ESM.docx]

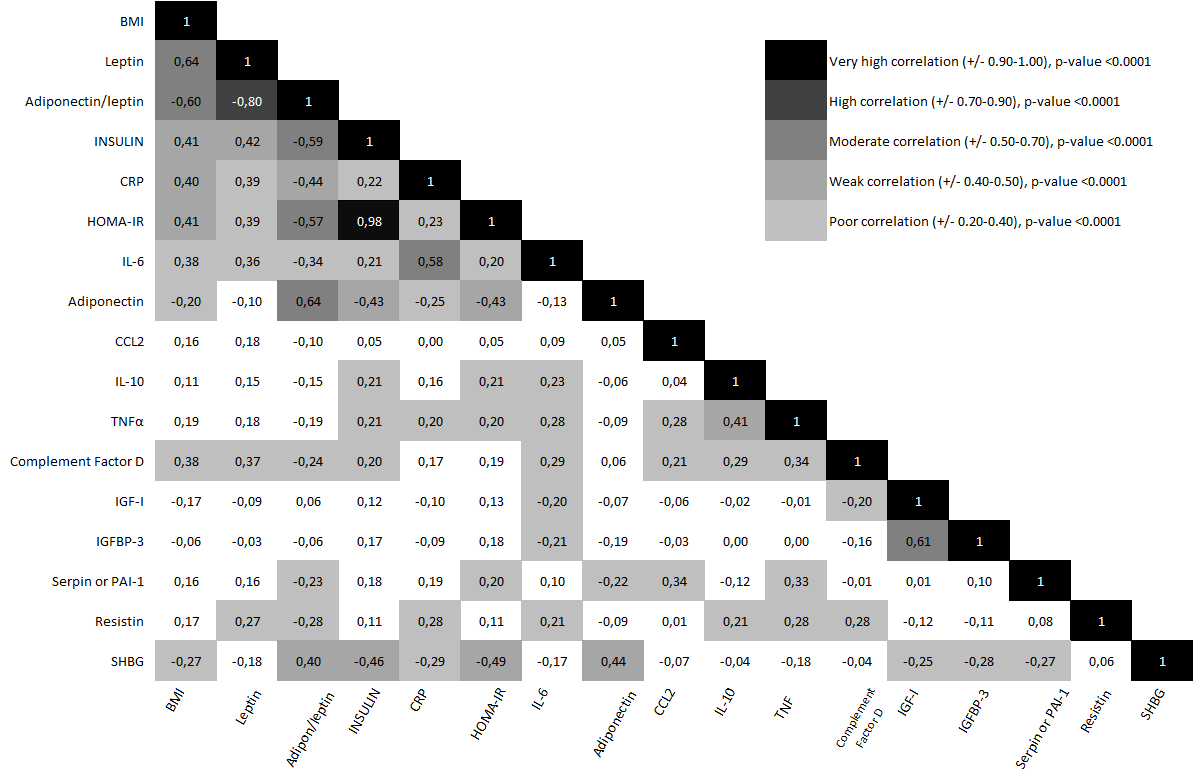


Supplementary Figure 1. Spearman rank’s correlation coefficients (rho) between concentrations of BMI, adipokines, metabolic, and inflammation biomarkers.
