# Supplementary material for: Effect of metformin and lifestyle intervention on adipokines and hormones in breast cancer survivors: a pooled analysis from two randomized controlled trials
Source: Breast Cancer Res Treat. 2024 Jan 26;205(1):49–59. doi: 10.1007/s10549-023-07241-2 (PMC11063007; doi:10.1007/s10549-023-07241-2)
Supplement: Supplementary file 2 — Supplementary file2 (DOCX 350 KB) [file 10549_2023_7241_MOESM2_ESM.docx]

**Supplementary Table S1 Baseline median and interquartile ranges of serum biomarkers by allocation arm**


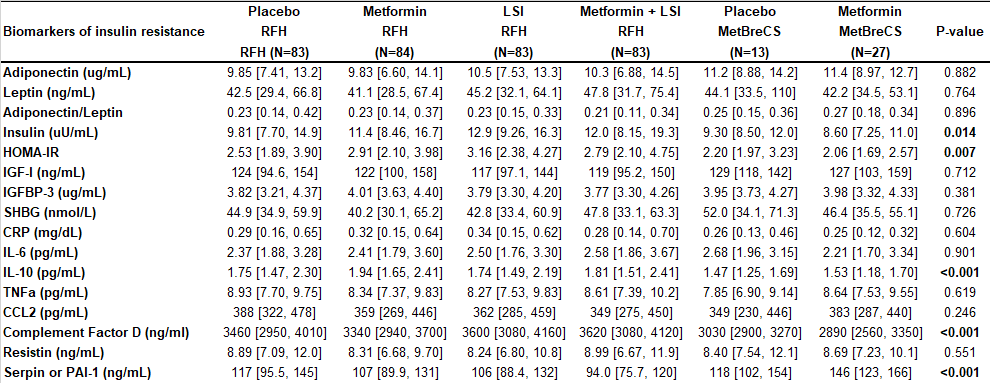


Lifestyle intervention (LSI)

P-values were derived from Kruskal-Wallis test comparing the six allocation arms.

**Supplementary Table S2 Baseline median and interquartile ranges of serum steroids by allocation arm**

| **Steroids** | **Placebo** | **Metformin** | **LSI** | **Metformin + LSI** | **Placebo** | **Metformin** | **P-value** |
| --- | --- | --- | --- | --- | --- | --- | --- |
|  | **RFH** | **RFH** | **RFH** | **RFH** | **MetBreCS** | **MetBreCS** |  |
|  | **(N=83)** | **(N=84)** | **(N=83)** | **(N=83)** | **(N=13)** | **(N=27)** |  |
| **Cortisol (nmol/L)** | 236 [198, 309] | 251 [194, 314] | 247 [202, 300] | 228 [186, 308] | 230 [191, 301] | 222 [169, 290] | 0.915 |
| **11-deoxycortisol (nmol/L)** | 0.60 [0.40, 0.80] | 0.50 [0.30, 0.80] | 0.60 [0.40, 0.80] | 0.50 [0.35, 0.80] | 0.50 [0.40, 0.70] | 0.50 [0.30, 0.90] | 0.839 |
| **17-hydroxyprogesterone (nmol/L)** | 0.40 [0.30, 0.60] | 0.40 [0.30, 0.60] | 0.40 [0.30, 0.50] | 0.40 [0.30, 0.60] | 0.40 [0.30, 0.60] | 0.60 [0.35, 0.80] | 0.323 |
| **Androstendione (nmol/L)** | 1.40 [1.10, 2.00] | 1.50 [1.00, 1.80] | 1.40 [1.10, 1.95] | 1.30 [1.10, 1.80] | 1.60 [1.30, 2.00] | 1.60 [1.25, 2.45] | 0.459 |
| **Testosterone (nmol/L)** | 0.60 [0.40, 0.70] | 0.50 [0.40, 0.70] | 0.60 [0.40, 0.80] | 0.60 [0.40, 0.80] | 0.70 [0.50, 0.80] | 0.60 [0.50, 0.70] | 0.580 |
| **Estradiol (pmol/L)** | 0.850 [0.850, 14.8] | 2.40 [0.850, 18.5] | 1.45 [0.850, 21.0] | 2.60 [0.850, 19.0] | 20.0 [15.0, 27.0] | 21.0 [16.5, 54.0] | **<0.001** |
| **Only women not taking AIs** | 17.0 [12.8, 33.0] | 20.0 [11.0, 27.0] | 22.0 [14.0, 30.0] | 19.0 [12.0, 31.0] | 20.0 [15.0, 27.0] | 21.0 [16.5, 54.0] | 0.520 |
| **Number of participants** | 32 | 35 | 39 | 37 | 13 | 27 |  |
| **Estrone (pmol/L)** | 5.30 [3.00, 73.8] | 7.15 [2.38, 91.5] | 4.40 [2.70, 104] | 5.80 [2.70, 89.0] | 85.0 [78.0, 102] | 100 [65.5, 131] | **<0.001** |
| **Only women not taking AIs** | 96.0 [63.5, 136] | 101 [64.0, 127] | 104 [72.5, 144] | 93.0 [50.0, 148] | 85.0 [78.0, 102] | 100 [65.5, 131] | 0.757 |
| **Number of participants** | 32 | 35 | 39 | 37 | 13 | 27 |  |

Lifestyle intervention (LSI)

P-values were derived from Kruskal-Wallis test comparing the six allocation arms.

**Supplementary Table S3 Baseline ranges of serum steroids in postmenopausal women not taking AIs, and absolute change at follow-up by main effect groups**
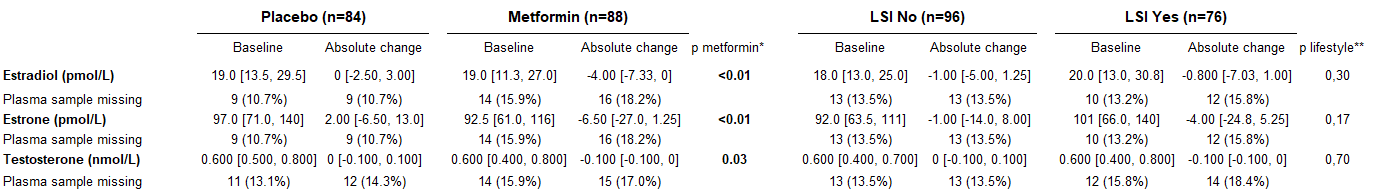


* p-value of the treatment covariate (Metformin vs Placebo) derived from a multivariate linear model fit on biomarker changes (Follow-up visit - Baseline), adjusted for the baseline value of the biomarker, study center, lifestyle intervention (LSI), age and baseline BMI.

** p-value of the LSI covariate (Yes vs No) derived from a multivariate linear model fit on biomarker changes (Follow-up visit - Baseline), adjusted for the baseline value of the biomarker, treatment, study center, age and baseline BMI.

**Supplementary Table S4 Biomarker changes according to estrogen receptor status and main effect groups**


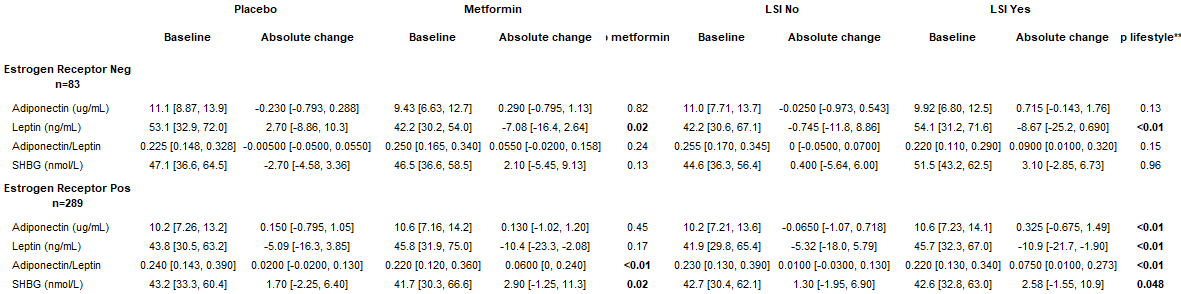


* p-value of the treatment covariate (Metformin vs Placebo) derived from a multivariate linear model fit on biomarker changes (Follow-up visit - Baseline), adjusted for the baseline value of the biomarker, study center, lifestyle intervention (LSI), age, baseline BMI and aromatase inhibitor therapy.

** p-value of the LSI covariate (Yes vs No) derived from a multivariate linear model fit on biomarker changes (Follow-up visit - Baseline), adjusted for the baseline value of the biomarker, treatment, study center, age, baseline BMI and aromatase inhibitor therapy.

**Supplementary Table S5. Participant baseline characteristics of the Italian and USA cohorts published (DOI 10.1186/s12967-022-03809-6)**
